# In-Vitro Efficacy of Cefiderocol in Carbapenem-Non-Susceptible Gram-Negative Bacilli of Different Genotypes in Sub-Region of North Rhine Westphalia, Germany

**DOI:** 10.3390/pathogens10101258

**Published:** 2021-09-29

**Authors:** Beniam Ghebremedhin, Parviz Ahmad-Nejad

**Affiliations:** Institute of Medical Laboratory Diagnostics, Center for Clinical and Translational Research, Helios University Clinic Wuppertal, Witten/Herdecke University, Department of Health, 42283 Wuppertal, Germany; parviz.ahmad-nejad@helios-gesundheit.de

**Keywords:** cefiderocol, siderophore, *Pseudomonas*, *Acinetobacter*, *Enterobacterales*, antimicrobial resistance

## Abstract

In the last two decades, the worldwide dissemination of multidrug-resistant Gram-negative bacteria (MDR-GNB) has continued. Therapy options for such infections caused by MDR-GNB remain scarce, and only few new antimicrobial agents have been granted market approval. Cefiderocol has been approved for the treatment of infections associated with aerobic GNB with limited therapy options. This study evaluated the in vitro efficacy of cefiderocol against carbapenem-non-susceptible clinical GNB isolates from Germany. A total of 115 non-duplicate carbapenem-nonsusceptible GNB isolates, 61 (53.05%) of which were *Enterobacterales* species and 54 (46.95%) were non-fermenters (*Acinetobacter baumanii* and *Pseudomonas aeruginosa*), were investigated for their cefiderocol susceptibility. Minimum inhibitory concentrations (MICs) for cefiderocol were determined by disk diffusion, according to EUCAST (European committee for antimicrobial susceptibility testing). Susceptibility rates were based on EUCAST breakpoints. In the absence of a species-specific breakpoint, pharmacokinetic/-dynamic breakpoints were used. The most common pathogen was *A. baumannii* (33.91%), followed by *Klebsiella pneumoniae* (31.3%), *P. aeruginosa* (13.04%) and *Escherichia coli* (9.57%). Overall, 83.6% (51/61) of the *Enterobacterales* and 81.48% (44/54) of the non-fermenters were susceptible towards cefiderocol. In total, 20 species of *Enterobacterales* and non-fermenting GNB were resistant towards cefiderocol, irrespective of the isolation year (2014 to 2021). Moreover, the majority of the resistant isolates were among the OXA-23 producing *A. baumannii* (*n* = 7/26; 26.92%) from patients hospitalized during 2018 and 2019. Cefiderocol demonstrated high in vitro susceptibility rates against a wide range of carbapenem-non-susceptible GNB, including carbapenemase-producing isolates. Cefiderocol exhibited stability against hydrolysis by all carbapenemases, including metallo-β-lactamases (MBLs), except that few OXA-producing isolates exhibited resistance towards cefiderocol.

## 1. Introduction

Infections due to multidrug-resistant Gram-negative bacteria (MDR-GNB), especially those associated with isolates which are non-susceptible towards carbapenem, have been very difficult to manage in the past two decades [1,2].

According to the World Health Organization (WHO), carbapenem-non-susceptible GNB (e.g., *Escherichia coli*, *Klebsiella pneumoniae*), including non-fermenters (e.g., *Acinetobacter baumannii*), are considered “critical” pathogens. The WHO declared the development of new antimicrobials against such MDR-GNB as a priority [3]; this is reasonable since there is an increasing number of MDR-GNB. This makes appropriate treatment difficult, resulting in high mortality rates [2,4,5,6,7]. According to the WHO, at least 700,000 people die each year due to MDR-related diseases, including 230,000 people who die due to MDR-associated tuberculosis [8]. Burnham et al. [9] re-estimated the deaths due to MDR-infections in the United States. In 2010, nearly two and a half million deaths occurred in the United States and approximately 70,000 of them died due to sepsis caused by MDR-GNB in US hospitals [9]. The authors conclude that their estimates for inpatient and outpatient deaths due to MDR-related infections gives more than 150,000 deaths, which is almost 7-fold higher than that reported by the Centers for Disease Control and Prevention (CDC: 23,000 deaths) in 2010 [9]. For the United States, the CDC reports that more than 35,000 people die out of >2.8 million MDR-related infections each year [10]. The European Centre for Disease Prevention and Control (ECDC) states on its website that 33,000 people die in Europe due to an infection associated with antibiotic-resistant bacteria each year [11]. This report coincides with a study by Cassini et al. [12]. According to Cassini et al. [12] MDR-GNB are responsible for approximately 33,000 deaths in Europe annually. However, there is an urgent need for better global surveillance and reporting mechanisms and databases for MDR-related infections and mortality to avoid or at least minimize controversies.

Among the recent antimicrobial agents, cefiderocol is the first siderophore cephalosporin which has been approved by the Food and Drug Administration (FDA) and European Medicines Agency (EMA), against different species of *Enterbacterales* and non-fermenting Gram-negative bacteria with different carbapenemase-types/sub-types that may have the potential to fill some of the remaining gaps in the treatment of MDR-GNB-associated infections [13,14,15,16,17].

The chlorocatechol side chain allows cefiderocol to bind extracellular free iron and to form Fe^3+^ chelate complex. This complex is formed by bacteria recognized as a siderophore and using active bacterial iron transport mechanisms as a “Trojan horse” through the outer membrane of GNB into the periplasmic space where it then binds to the penicillin-binding protein 3 (PBP-3) and thus inhibits the synthesis of peptidoglycan bacterial cell wall which leads to lysis and death of the bacteria [13].

Cefiderocol shows stability against numerous β-lactamases of Ambler classes A to D, including carbapenemases and thus exhibits activity against *Enterobacterales*, *Pseudomonas aeruginosa*, the *A. baumannii* complex, but also against some other GNBs [13,14]. There is no effectiveness against Gram-positive pathogens (such as staphylococci, streptococci or enterococci), anaerobes and intracellular bacteria, e.g., *Mycoplasma pneumoniae* [15]. Additionally, and similar to the oxyimino-β-lactams, ceftazidime and cefepime, the stability of cefiderocol to β-lactamases and activity against GNB, including *P. aeruginosa*, is enhanced via the pyrrolidinium group on the C3 side chain and the carboxypropyl-oxyimino group on the C7 side chain, which also enhances cefiderocols’s stability towards β-lactamases [16]. Cefiderocol appears to be well positioned to cover the increasing number of infections caused by carbapenem-resistant and MDR-GNB, including ESBL- and carbapenemase-producing isolates [15]. Hence, we investigated different carbapenem-non-susceptible MDR-GNB *Enterobacterales* species and non-fermenting GNB, *P. aeruginosa*, and *A. baumannii* complex for their susceptibility towards cefiderocol.

## 2. Results

The cefiderocol MIC levels were highly variable depending on the GNB species, with an overall MIC range of ≤0.03 to 24 mg/L. The species *E. coli*, *C. freundii*, and *S. marcescens* uniformly displayed 100% susceptibility to cefiderocol, regardless of the harbored carbapenemase genes (Table 1). When the EUCAST non-species-specific pharmacokinetic-pharmacodynamic (PK-PD) breakpoints were applied for *A. baumannii*, both *A. baumannii* and *K. pneumoniae* had resistance rates of 23.1% and 19.4%, respectively, depending on the resistance genes they harbored. In contrast to *A*. *baumannii*, only 6.7% of the *P. aeruginosa* isolates were resistant towards cefiderocol.

### 2.1. Enterobacterales

The mean susceptibility rate of all *Enterobacterales* species towards cefiderocol was approximately 81%. Among the surveillance isolates gathered in 2014–2021 (Table 1), all *E. coli* isolates (*n* = 11) had cefiderocol MICs ≤2 mg/L. The mean cefiderocol MIC was 0.25 mg/L. This applies also for the two *S. marcenscens* isolates with cefiderocol MICs of 0.016 and 0.25 mg/L, and the single *R. ornithinolytica* isolate with an MIC value of 0.016 mg/L, respectively. Similarly, all three *C. freundii* isolates had cefiderocol MICs ≤2 mg/L. The same is true for three *E. cloacae* isolates (*n* = 3/4) with MICs ≤2 mg/L, exlusively one *E. cloacae* (*n* = 1/4) which was resistant towards cefiderocol with MIC value of 9 mg/L. This isolate originated from a clinical sample in 2021 and co-carried VIM-1 and OXA-48 genes. We identified a single *Klebsiella aerogenes* isolate with a relatively low cefiderocol MIC value of 0.064 mg/L. The second *K. aerogenes* isolate (isolation year 2019) was inhibited at cefiderocol MIC = 16 mg/L without carrying any carbapenemase gene, but AmpC gene.

As for the 36 investigated *K. pneumoniae* isolates, 3 of 5 KPC-2 (*n* = 3/5) and all 4 KPC-3 (*n* = 4/4) producing isolates were cefiderocol-susceptible, with MIC values ranging from 0.023 and 2 mg/L and 0.064 and 2 mg/L, respectively. All OXA-48 producing *K. pneumoniae* isolates (*n* = 12/12) were cefiderocol-susceptible with MIC values ≤0.5 mg/L. Few *K. pneumoniae* isolates harboring the carbapenemase genes OXA-181 (*n* = 2/2), OXA-204 (*n* = 1/1), OXA-232 (*n* = 1/1), VIM-1 (*n* = 1/1), and VIM-2 (*n* = 1/1) uniformly displayed 100% susceptibility towards cefiderocol with MIC values ≤ 0.032.

However, the overall susceptibility rate was 80.6% for all the *K. pneumoniae isolates* irrespective of their resistance *bla* genes, defining this group as the most cefiderocol-resistant within the *Enterobacterales*.

### 2.2. P. aeruginosa and A. baumannii

There were 15 isolates of *P. aeruginosa* gathered from 2014 to 2021 (Table 1), and 93.3% had cefiderocol MIC ≤2 mg/L. The β-lactamase gene detected within these isolates was *bla*_VIM-2_ β-lactamase (*n* = 9/15), all were cefiderocol-susceptible within the MIC range of 0.19 and 2 mg/L.

There were 39 isolates of *A. baumannii* gathered from 2014 to 2021, and 77.9% had cefiderocol MICs ≤2 mg/L. When the EUCAST non-species-specific pharmacokinetic-pharmacodynamic (PK-PD) breakpoints were applied for *A. baumannii*, 23.1% of the carbapenem-resistant *A. baumannii* isolates exhibited resistance towards cefiderocol with MIC >2 to 16 mg/L, especially those carrying the *bla*_OXA-23-type_ β-lactamase. Among the isolates harboring this gene 29.1% (*n* = 7/24) were resistant towards cefiderocol with MIC values ranging from 3 to 24 mg/L (Table 2).

Table 1 summarizes the in vitro activity of cefiderocol against carbapenemase-producing isolates and carbapenemase-negative isolates of carbapenem-non-susceptible *Enterobacterales* and carbapenem-non-susceptible *P. aeruginosa* and *A. baumannii*.

### 2.3. Cefiderocol-Susceptible Enterobacterales, P. aeruginosa and A. baumannii with Emphasis on bla_OXA-23-type_ and bla_OXA-48-type_ β-lactamase

To emphasize the high MIC levels for cefiderecol in particular isolates among the susceptible GNB species with various carbapenemase genes, we selected the predominant species with their harbored genes in Table 3. This table indicates that *A. baumannii* isolates carrying the *bla*_OXA-23-type_ β-lactamase show a tendency for higher MIC levels >0.125 to 2 mg/L, with the emphasis of MIC frequency of 0.19. Whereas the *K. pneumoniae* isolates carrying the *bla*_OXA_-_48-type_ β-lactamase displayed frequently lower MIC levels for cefiderocol: *n* = 7 of 12 isolates with MIC levels 0.023 mg/L and 0.047 mg/L. The same applies for *E. coli* isolates carrying the *bla*_OXA-48-type_ β-lactamase, at least for *n* = 3/3 isolates with MIC = 0.016 and 0.125 mg/L).

## 3. Discussion

Cefiderocol, an innovative parenteral siderophore cephalosporin, has demonstrated potent bactericidal efficacy against Gram-negative bacilli (GNB) such as carbapenem-non-susceptible *Enterobacterales* species, *Pseudomonas aeruginosa*, and *Acinetobacter baumannii* that are multidrug-resistant (MDR) and producers of diverse carbapenemases and other β-lactamases.

Its efficacy has been investigated in large international surveillance studies carried out since 2014 (SIDERO-WT studies), covering over 28,000 Gram-negative bacteria. Overall, the MIC_90_ for *Enterobacterales* (including *Escherichia coli*, *Klebsiella* spp., *Citrobacter* spp., *Enterobacter* spp., *Serratia* spp. and other species) ranged from 0.25 to 1 mg/L, with no significant geographical or temporal differences. The overall activity of cefiderocol against *Enterobacterales* from surveillance studies revealed that >98% of isolates were inhibited at concentrations of 2 mg/L. However, this was retained against most isolates resistant to expanded-spectrum cephalosporins and carbapenems, including those producing different types of serine carbapenemases and metallo-β-lactamases. Naas et al. [17] evaluated the in vitro activity of cefiderocol and comparators against Gram-negative bacilli (GNB) clinical isolates from France. Cefiderocol demonstrated high in vitro susceptibility rates against a wide range of GNB, including meropenem-resistant strains, and was significantly more active than comparators against pneumonia isolates. Overall, 99.0% *Enterobacterales* and (99.7%) non-fermenters were cefiderocol susceptible, including 100% of meropenem-resistant *S. maltophilia* and *P. aeruginosa* isolates. In contrast to the findings of Naas et al. [17], the cefiderocol susceptibility rate of our isolates was lower for both GNB groups, the *Enterobacterales* and the non-fermenters. Our results are consistent with previous reports by Mushtaq et al. [18], at least for *Enterobacterales* and *A. baumannii.* The overall efficacy rates of cefiderocol in our carbapenem-non-susceptible GNB isolates were: 81.9% for *Enterobacterales* species, 93.3% *P. aeruginosa*, and the lowest rate of 76.9% for *A. baumannii.*

Mushtaq et al. [18] evaluated cefiderocol MICs against GNB by use of iron-depleted Mueller–Hinton broth, the gold standard method. The panel comprised 305 isolates of *Enterobacterales*, 111 of *P. aeruginosa*, and 99 of *A. baumannii*, and all were carbapenem-non-susceptible and MDR to other agents. Of all *Enterobacterales*, 78.7% were inhibited at 2 mg/L cefiderocol, with rates of 80 to 100% for isolates with all modes of carbapenem resistance except NDM enzymes (41.0% inhibited at 2 mg/L) or combinations of ESBL and other mechanisms (61.5% inhibited at 2 mg/L). Cefiderocol inhibited 81.1% of all *P. aeruginosa* isolates at 2 mg/L, with rates of 80 to 100% for isolates with VIM, IMP, GES, or VEB β-lactamases and slightly lower rates for those with NDM (45.5% at 2 mg/L) and PER (66.7% at 2 mg/L) enzymes. 80.8% of the *A. baumannii* isolates were inhibited at 2 mg/L, with rates of >85% for isolates with OXA-51-like, -23, -24, or -58 enzymes and 50% at 2 mg/L for those with NDM carbapenemases. In our study, 29.2% of the OXA-23 producing *A. baumannii* isolates were resistant towards cefiderocol at MIC >2 to 24 mg/L or at <17 mm of inhibition zone diameter. However, Malik et al. [19] examined the resistance mechanisms in 12 *A. baumannii* isolates with cefiderocol MICs ranging from ≤0.03 to 32 mg/L. Their resistance to this new agent could not be explained by any β-lactamase activity.

Iregui et al. [20] determined higher cefiderocol MICs in endemic *E. coli* and *K. pneumoniae* isolates at the Medical Centers in New York City that were cephalosporin resistant. The activity of cefiderocol has been reported to be maintained in *Enterobacterales* possessing a wide variety of carbapenemases, including KPC, NDM VIM, IMP, and OXA-48. Of the 78 carbapenem-resistant *A. baumannii* isolates, including 47 with OXA-23-type β-lactamases, the MIC_90_ value was 8 mg/L, which was identical to the subset of isolates with OXA-23-type β-lactamases. Moreover, Malik et al. [19] conclude that the cefiderocol resistance in *A. baumannii* isolates was associated with reduced expression of the siderophore receptor gene *pirA.* Mutations involving PBP3 may have contributed to resistance in one isolate. Future studies should investigate the role of the siderophore receptors. Poirel et al. [21] investigated a series of *A. baumannii* clinical isolates with elevated MICs of cefiderocol and showed that PER-like β-lactamases, and to a lesser extent NDM-like β-lactamases, significantly contributed to reduced susceptibility towards cefiderocol.

Furthermore, Jacobs et al. [22] revealed higher MICs for cefiderocol in *Enterobacterales* possessing an ESBL, carbapenemase, or AmpC-type β-lactamase compared with isolates lacking these enzymes.

Moreover, Ito et al. and Ito-Horiyama et al. [23,24] investigated the mechanism of action of cefiderocol has revealed that the specific outer membrane iron transporters CirA and Fiu in *E. coli* and PiuA in *P. aeruginosa* are involved in the active transport of cefiderocol. Such mutations in GNB resulted in deficiency in the activity of these transporter molecules and revealed significantly ≥16-fold increased MICs of cefiderocol. However, experiments with purified enzyme extracts of frequent carbapenemases, e.g., KPC-3, IMP-1, VIM-2, NDM-1, OXA-48, OXA-23 β-lactamases, revealed that cefiderocol remained stable when exposed to these enzymes [23,24,25].

In this study, we found that NDM-1/-6 positive isolates (*n* = 4 of 4, 100%) were inhibited by ≤2 mg/L cefiderocol. However, this was not true for such isolates which were positive for NDM-5/-20, NDM-7/NDM-19, and NDM-9. These carbapenemase genes encoding for New Delhi metallo-β-lactamase were harbored by *A. baumannii* (*n* = 1) and *K. pneumoniae* isolates (*n* = 2) exhibiting MIC levels >16 mg/L of cefiderocol. The isolates were gained from clinical specimens during 2014 to 2017. Furthermore, Kazmierczak et al. [15] found that 35.7% of the NDM-positive isolates were not inhibited by ≤4 mg/L cefiderocol. The authors received these *K. pneumoniae* isolates from Turkey with a cefiderocol MIC level of 8 mg/L that carried NDM-1 and a CTX-M-1-type ESBL. According to their sequence analysis of the porin gene, one isolate had lost OmpK35 and three others were expected to produce truncated OmpK36 proteins of different lengths. These are findings which underscore the regional or intercontinental diversity of strains with non-susceptibility towards cefiderocol.

The previously approved antimicrobial agents are characterized by a good effect on MDR-GNB. Resistance to these antimicrobials is currently still rare, but has already emerged partially before their approval, e.g., cefiderocol, as observed in the bacterial population of the current study. Due to their very sufficient efficacy towards various MDR-GNB, these antimicrobials should not be used as new broad-spectrum antibiotics in calculated therapy—despite the broad spectrum of activity against MDR-GNB. The current study examined the activity of cefiderocol, a promising parenteral siderophore cephalosporin with time-dependent bactericidal activity against GNB, especially those in late-stage clinical development for treatment of infections caused by MDR-GNB. The distribution of carbapenemase types varies among isolates in our region and other regions, or even continental regions, which underscores the importance of knowing the local incidence of different resistance mechanisms when evaluating treatment options. Regardless of GNB species, cefiderocol strongly demonstrated sufficient in vitro activity towards carbapenemase-producing isolates. Cefiderocol represents an addition to the limited last resort of existing antimicrobial agents available for treatment of severe infections caused by MDR-GNB.

We believe, due to the heterogeneity of carbapenem-non-susceptible MDR-GNB, that in order to maintain the effectiveness of this reserve antimicrobial agent for as long as possible, it should only be administered if an additional benefit compared to established agents has been proven, e.g., via antimicrobial susceptibility testing. The therapy options for infections with MDR-GNB should be standardized within the local clinic in order to avoid inefficient therapies as best as possible.

This study included a relatively small number of isolates, particularly for any individual species. Furthermore, isolates were derived from a single region, which may limit the generalizability of our findings to other regions with differences in the composition of common Gram-negative resistance mechanisms. Though our region is a rather low-prevalence area for carbapenemase-producing GNB, the current results need to be verified in a larger study that includes geographically diverse isolates with higher frequency of different *bla* genes.

## 4. Materials and Methods

One-hundred fifteen non-duplicate clinical GNB isolates of the years 2014 to 2021 (Table 4) were identified using matrix-assisted laser-desorption ionization time-of-flight mass spectrometry (MALDI-TOF-MS; Bruker Daltonics, Bremen, Germany). AST results were determined using the BD Phoenix automated system (BD Diagnostics, Heidelberg, Germany).

These isolates had been collected in various surveillance studies performed in our Helios hospitals in North Rhine Westphalia between 2014 and 2021 and either characterized in our molecular laboratory or were sent to the German National Reference Centre for Multidrug-Resistant Gram-negative Bacteria for molecular typing Reference strains for verification. *Escherichia coli* DSM 1103 and *Pseudomonas aeruginosa* DSM 1117 were used for quality control. For all isolates with resistance to third-generation cephalosporins and/or carbapenems, the presence of beta-lactamase genes was tested via Real-Time-PCR/sequencing, as previously described [26,27,28,29,30,31].

Antimicrobial susceptibility testing (AST) was interpreted according to EUCAST clinical breakpoints version 11.0 EUCAST, www.eucast.org (accessed on 31 August 2021), (Table 5), [31]. AST for cefiderocol was determined by epsilon test (Bestbion/Liofilchem, Cologne, Germany) on Mueller–Hinton agar according to the manufacturer’s protocol. From an overnight agar plate, well-isolated colonies were suspended in saline to achieve a 0.5 McFarland standard turbidity. By use of a sterile cotton swab, the inoculum was streaked over the entire area of the Mueller–Hinton (MH) agar plate. Thereafter, the test strip was firmly applied to the surface of the inoculated agar plate and incubated at 35 ± 1 °C for 16–20 h. According to the manufacturer’s instruction for reading the plates after the required incubation period, and only when an even lawn of growth was distinctly visible, the MIC value was read where the relevant inhibition ellipse intersects the strip. For bactericidal antimicrobials like cefiderocol, the MIC endpoint should be read at complete inhibition of growth. Haze and macro- or micro-colonies within 3 mm from the strip should be read as growth (Technical sheet from Bestbion/Liofilchem, Cologne, Germany).

Growth along the entire gradient i.e., no inhibition ellipse, indicates that the value is greater than or equal to (≥) the highest value on the scale. An inhibition ellipse that intersects below the lower end of the scale is read as less than (<) the lowest value. Intersection between two scale segments should be rounded up to the higher value. An MIC of 0.125 mg/L is considered the same as 0.12 mg/L for reporting purposes (Technical sheet from Bestbion/Liofilchem, Cologne, Germany).

The European Committee on Antimicrobial Susceptibility Testing (EUCAST) has set cefiderocol breakpoints with MIC values of ≤2 mg/L and >2 mg/L for susceptible and resistant categories, respectively, for *Enterobacterales* and *Pseudomonas* spp., and also pharmacokinetic/pharmacodynamic (PK/PD) breakpoint MIC values of ≤2 mg/L for susceptibility, while a breakpoint was not set for *A. baumannii* due to insufficient clinical evidence. However, *A. baumannii* was susceptible if inhibition zone diameter for cefiderocol disk was >17 mm [31].

## Figures and Tables

**Table 1:** In vitro activity of cefiderocol against carbapenem-resistant *Enterobacterales* and carbapenem-non-susceptible *P. aeruginosa* and *A. baumannii* isolates producing various β-lactamases (total *n* = 115).

	MIC in mg/L																								
GNB Species	0.016	0.023	0.032	0.047	0.064	0.094	0.125	0.19	0.25	0.38	0.39	0.5	0.75	1	1.5	2	3	4	8	9	12	16	17	24	Total (Species)
*A. baumannii*	2					1	1	5	2	5	1	2	3	3	3	2	1	2	1		2	2		1	39
*C. freundii*	1														1	1									3
*E. cloacae*					1										2					1					4
*E. coli*	4						1		1	2				1	1	1									11
*K. aerogenes*			1																			1			2
*K. oxytoca*	1																	1							2
*K. pneumoniae*	4	5	2	4	2		3		2	2		3				2	4					2	1		36
*P. aeruginosa*								2	2			3	1	1	1	4	1								15
*R. ornithinolytica*	1																								1
*S. marcescens*	1								1																2
total (isolates)	14	5	3	4	3	1	5	7	8	9	1	8	4	5	8	10	3	3	1	1	2	5	1	1	115

**Table 2:** A subset of cefiderocol-resistant and carbapenem-non-susceptible *Enterobacterales, P. aeruginosa* and *A. baumannii* isolates producing various β-lactamases, *n* = 20; R%, resistance rate in %.

	MIC mg/L Cefiderocol									
	3	4	8	9	12	16	17	24		
GNB Species									*n*	R%
*A. baumannii*	1	2	1		2	2		1	9	23.1%
*NDM-1/-6 + OXA-23*		1							1	
*NDM-9*						1			1	
*OXA-23*	1	1	1		2	1		1	7	
*E. cloacae*				1					1	25.0%
*VIM-1/OXA-48*				1					1	
*K. aerogenes*						1			1	50.0%
*AmpC*						1			1	
*K. oxytoca*		1							1	50.0%
*VIM-4*		1							1	
*K. pneumoniae*	4					2	1		7	19.4%
*ESBL*	2								2	
*KPC-2*	1					1			2	
*NDM-1/NDM-6/NDM-16/OXA-48*	1								1	
*NDM-5/-20*							1		1	
*NDM-7/NDM-19*						1			1	
*P. aeruginosa*	1								1	6.7%
*AmpC*	1								1	
total	6	3	1	1	2	5	1	1	20	

**Table 3:** A selection of cefiderocol-susceptible and carbapenem-non-susceptible *Enterobacterales*, *P. aeruginosa* and *A. baumannii* isolates producing various β-lactamases—with emphasis on *bla*_OXA-23-type_ and *bla*_OXA-48-type_ β-lactamase, *n* = 84.

	MIC mg/L Cefiderocol																
GNB Species, Genes	0.016	0.023	0.032	0.047	0.064	0.094	0.125	0.19	0.25	0.38	0.39	0.5	0.75	1.0	1.5	2.0	*n*
*A. baumannii*	2					1	1	5	2	5	1	2	3	3	3	2	30
*GIM-1*														1			1
*NDM-1/-6*	1									1							2
*NDM-1/-6 + OXA-23*																1	1
*NDM-2*															1		1
*OXA-164*						1											1
*OXA-23*							1	5	1	2	1	2	2	2	1		17
*OXA-40*	1																1
*OXA-58*													1			1	2
*OXA-72*									1	2					1		4
*E. coli*	4						1		1	2				1	1	1	11
*KPC-2*										1							1
*KPC-3*	2																2
*NDM-3*									1								1
*NDM-5*															1	1	2
*OXA-181*										1				1			2
*OXA-48*	2						1										3
*K. pneumoniae*	4	5	2	4	2		3		2	2		3				2	29
*ESBL + other mechanisms*																1	1
*KPC-2*		1		1						1							3
*KPC-3*					1				1			1				1	4
*NDM-1/-6*	1																1
*NDM-1/NDM-6/NDM-16*												1					1
*OXA-181*			1						1								2
*OXA-181/OXA-48*					1												1
*OXA-204*	1																1
*OXA-232*										1							1
*OXA-48*	2	4	1	3			1					1					12
*VIM-1*							1										1
*VIM-2*							1										1
*P. aeruginosa*								2	2			3	1	1	1	4	14
*AmpC + other mechanisms*												1		1	1	2	5
*VIM-2*								2	2			2	1			2	9
total isolates	10	5	2	4	2	1	5	7	7	9	1	8	4	5	5	9	84

**Table 4:** The bacterial population which was investigated for cefiderocol susceptibility. *A. baumannii*, *E. coli*, and *K. pneumoniae* producing various β-lactamases and other resistance mechanisms were the predominant species among the total number of the isolates, *n* = 115.

GNB Species, Harbored Genes	*n*	% of Total Isolates
*A. baumannii*	39	33.91%
*GIM-1*	1	
*NDM-1/-6*	2	
*NDM-1/-6 + OXA-23*	2	
*NDM-2*	1	
*NDM-9*	1	
*OXA-164*	1	
*OXA-23*	24	
*OXA-40*	1	
*OXA-58*	2	
*OXA-72*	4	
*C. freundii*	3	2.61%
*AmpC*	1	
*OXA-162*	1	
*VIM-1*	1	
*E. cloacae*	4	3.48%
*NDM-1/-6*	1	
*VIM-1*	1	
*VIM-1/OXA-48*	1	
*VIM-4*	1	
*E. coli*	11	9.57%
*KPC-2*	1	
*KPC-3*	2	
*NDM-3*	1	
*NDM-5*	2	
*OXA-181*	2	
*OXA-48*	3	
*K. aerogenes*	2	1.74%
*AmpC*	1	
*OXA-48*	1	
*K. oxytoca*	2	1.74%
*KPC-2*	1	
*VIM-4*	1	
*K. pneumoniae*	36	31.30%
*ESBL*	3	
*KPC-2*	5	
*KPC-3*	4	
*NDM-1/-6*	1	
*NDM-1/NDM-6/NDM-16*	1	
*NDM-1/NDM-6/NDM-16/OXA-48*	1	
*NDM-5/-20*	1	
*NDM-7/NDM-19*	1	
*OXA-181*	2	
*OXA-181/OXA-48*	1	
*OXA-204*	1	
*OXA-232*	1	
*OXA-48*	12	
*VIM-1*	1	
*VIM-2*	1	
*P. aeruginosa*	15	13.04%
*AmpC*	6	
*VIM-2*	9	
*R. ornithinolytica*	1	0.87%
*KPC-2*	1	
*S. marcescens*	2	1.74%
*OXA-48*	1	
*VIM-1*	1	
total	115	

**Table 5:** EUCAST has values of S ≤ 2 mg/L and R > 2 mg/L for *Enterobacterales* and *P. aeruginosa*—EUCAST, European Committee on Antimicrobial Susceptibility Testing; S, susceptible; R, resistant. §: Non-species-specific pharmacokinetic-pharmacodynamic (PK-PD) breakpoint. #: EUCAST provided disk correlates associated with the susceptible PK-PD breakpoint for *A. baumannii*.

Gram-Negative Bacilli	MIC (mg/L)		Inhibition Zone Diameter (mm)	
	S	R	S	R
*Enterobacterales* spp.	≤2	>2	≥22	<22
*Pseudomonas aeruginosa*	≤2	>2	≥22	<22
*Acinetobacter baumannii*	≤2 ^§^	>2 ^§^	≥17 ^#^	

## Data Availability

The data presented in this study are available upon reasonable request from the corresponding author via e-mail.
